# Beer's Law‐Why Integrated Absorbance Depends Linearly on Concentration

**DOI:** 10.1002/cphc.201900787

**Published:** 2019-10-10

**Authors:** Thomas G. Mayerhöfer, Andrei V. Pipa, Jürgen Popp

**Affiliations:** ^1^ Spectroscopy/Imaging Leibniz Institute of Photonic Technology Albert-Einstein-Str. 9 D-07745 Jena Germany; ^2^ Plasmadiagnostics Leibniz Institute for Plasma Science and Technology Felix-Hausdorff-Str. 2 D-17489 Greifswald Germany; ^3^ Spectroscopy/Imaging Leibniz Institute of Photonic Technology Albert-Einstein-Str. 9 D-07745 Jena Germany; ^4^ Institute of Physical Chemistry and Abbe Center of Photonics Friedrich Schiller University Helmholtzweg 4 D-07745 Jena Germany

**Keywords:** absorbance, Beer's law, concentration dependence, dispersion analysis, isotropic media

## Abstract

As derived by Max Planck in 1903 from dispersion theory, Beer's law has a fundamental limitation. The concentration dependence of absorbance can deviate from linearity, even in the absence of any interactions or instrumental nonlinearities. Integrated absorbance, not peak absorbance, depends linearly on concentration. The numerical integration of the absorbance leads to maximum deviations from linearity of less than 0.1 %. This deviation is a consequence of a sum rule that was derived from the Kramers‐Kronig relations at a time when the fundamental limitation of Beer's law was no longer mentioned in the literature. This sum rule also links concentration to (classical) oscillator strengths and thereby enables the use of dispersion analysis to determine the concentration directly from transmittance and reflectance measurements. Thus, concentration analysis of complex samples, such as layered and/or anisotropic materials, in which Beer's law cannot be applied, can be achieved using dispersion analysis.

## Graphical abstract

**Absorbance** at a particular spectral point does not necessarily depend linearly on the concentration. Based on electromagnetic theory, it can be demonstrated, however, that for the integrated absorbance, Beer's law still holds.

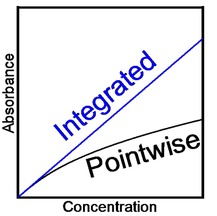

In his seminal 1852 paper, Beer showed that the transmittance of light through a cuvette is constant within experimental error when the product of the thickness of the cuvette times the concentration of the absorber is constant.^1^ This empiric law was examined by Max Planck in 1903 based on his dispersion theory.^2^ Planck showed that Beer's findings were valid only for spectrally narrow and weak absorption bands and that the absorption maximum shifts with an increasing number density, or concentration, of oscillators. Planck's dispersion theory considered local field effects, which he did not investigate separately from the influence of the quadratic dependence of the complex index of refraction from the dielectric function following from Maxwell's wave equation. This quadratic dependence, and more fundamentally, the concept of a dielectric function were still under investigation in 1930.^3^ Planck's results were included, e. g., in Kayser's Handbook of Spectroscopy (vol. 4), which was a reference for spectroscopy at that time.^4^ Planck's finding is also implicitly contained in Max Born's book “Optik”.^5^ In fact, Born presented the Clausius‐Mosotti and the Lorentz‐Lorenz equations in his book. If local field effects had been disregarded and the index of refraction as well as the dielectric constant considered complex functions of the frequency, then a connection to Beer's law and the concentration dependence of absorbance could have been established. Born's book, however, neither mentioned nor discussed Beer's law or Planck's paper. Unfortunately, when Born's book was translated into English, the contents of the last two chapters about molecular optics and emission, absorption and dispersion were mostly omitted.^6^

In the early 1960s, the connection between dispersion theory and Beer's law was known.^7^ However, due to the use of the simplification introduced by Lorentz in 1906^8^ known as the Lorentz‐profile,^9^ instead of a damped harmonic oscillator (“Lorentz‐oscillator”), a linear dependence of absorbance from concentration was found, which indicated that Planck's original findings had been lost.

Absorbance was not commonly used before approximately 1910, which is probably why Planck did not directly derive the concentration dependence of absorbance and the formulation known today as Beer's law. Accordingly, absorbance is not mentioned in vol. 3 (published 1903) of Kayser's handbook in its discussion of the numerous forms of absorption laws common at that time.^10^ The origin of this quantity has been attributed to a suggestion that was made in 1900^11^ and used afterwards.^12^ Thus, the absorbance A is given by Equation 1:
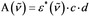



where $\epsilon^{*}\left(\tilde{v}\right)$ is the molar attenuation coefficient, *c* is the concentration and *d* is the sample thickness. A well‐known limitation of Beer's law is that monochromatic light must be used, since, as eqn. (1) implies, it holds for every spectral point, but molar attenuation coefficients are often given only for the peak frequency or wavenumber of a band and not in a frequency‐dependent form.

Additional well‐known limitations are that chemical interactions between two molecules can alter the molar attenuation coefficients and that instrumental factors such as finite spectral resolution and deviations of the detector from linearity can invalidate the results from eqn. (1). Contemporary textbooks do not address the possibility that the linear concentration dependence could be fundamentally incorrect.^13^, ^14^ Reviews of deviations from Beer's law and tutorials on Beer's law as well as spectra processing and analysis do not even mention this fundamental limitation.^15^, ^16^, ^17^, ^18^ The current literature on the correction of “artifacts” and deviations from Beer's law relies on the additivity and general linearity of absorbance.^19^, ^20^, ^21^, ^22^, ^23^

In the following, we will briefly introduce why absorbance is not linearly dependent on the concentration, even in the absence of any interactions. More important, we will show how determining either the integrated absorbance of a band or the classical oscillator strength, instead of the absorbance at a certain spectral point or the peak absorbance, can overcome the corresponding limitation.

We recently derived the concentration dependence of absorbance from dispersion theory.^24^ We also derived this dependence from simple electromagnetic theory,^25^ without referring to a particular oscillator model or the corresponding shape functions.

Assuming that there are no interactions between dipoles, i. e., no chemical interactions and no local field effects,^26^ the following expression for the relative dielectric function *ϵ_r_* is obtained [Eq. 2]:^25^

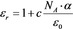



where *c* is the molar concentration, *N_A_* is Avogadro's constant, *α* is the (complex) polarizability and $\epsilon_{0}$ is the permittivity of free space.

In addition to chemical interactions and local field effects, nearfield interactions and electromagnetic coupling, which were recently shown to influence the complex index/indices of refraction and cause nonadditivity of the absorption cross sections,^27^ are explicitly excluded. Thus, the medium, i. e., the sample, is assumed to be isotropic (scalar dielectric function), not only isotropic in relation to the wavelength but completely homogenous. Under these very restrictive conditions, Maxwell's wave equation leads to the simple result that the relative dielectric constant (*n=*n+*i*k) equals the index of refraction squared, $\epsilon_{r}=n^{2}$ . Employing this relation, we find that [Eq. 3]:
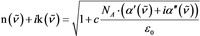



In eqn. (3), $n(\tilde{v})$ and $k(\tilde{v})$ are the index of refraction and the index of absorption. Likewise, $\alpha^{'}\left(\tilde{v}\right)$ and $\alpha^{''}\left(\tilde{v}\right)$ are the real and the imaginary parts of the polarizability.

Further investigation of the relation for the imaginary part of the dielectric function,$\epsilon_{r}^{''}$ (eqn. (2)), reveals that the change of this imaginary part is clearly linearly dependent on the concentration for both a single spectral point and the integration over a spectral region. The reason for this relationship is that the concentration is not dependent on the wavenumber or frequency and can simply be extracted from the integral [Eq. 4]:
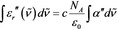



Because the absorbance is connected with the index of absorption via Equation 5:
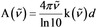



we need to examine the concentration dependence of the index of absorption more closely to understand how absorbance depends on the concentration under the constraints above.

Based on eqn. (3), the absorbance does not depend linearly on the concentration. However, we can apply the same approximation that led to the derivation of the Lorentz‐profile from the Lorentz‐oscillator, namely, that for *x*≪1, $\sqrt{1+x}\approx 1+x/2$ :^9^

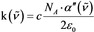



Under this constraint, which implies that the peak value of $\alpha^{''}\left(\tilde{v}\right)$ , and thus the absorption, is small, we immediately see that, as in eqn. (4), evaluation of the concentration dependence at a certain spectral point and integration of the absorbance over a band yield the same result.

If we use the exact relation between k and the real and imaginary parts of the dielectric function [Eq 7]:
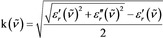



and replace the dielectric function based on Equation (2), the result is no longer linearly dependent on the concentration [Eq. 8]:
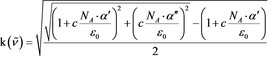



This can be further illustrated by using the damped harmonic oscillator model and employing one oscillator to describe the polarizability in a certain spectral range. The corresponding relative dielectric function is given by Equation 9:^24^, ^25^

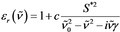



where *S* ^*2^ is the molar oscillator strength, ${\tilde{v}}_{0}$ is the oscillator position and γ is the damping constant.

We use eqs. (7)–(9) to calculate k and eqn. (5) to calculate the absorbance A. From these equations, it is not obvious that the resulting band shapes change with concentration, in contrast to those of Lorentz profiles. Planck showed this relationship by calculating different limiting values using a slightly different oscillator model that included a local field correction.^2^ Here, we use a more illustrative method and consider that the imaginary part of the relative dielectric function is given by $\epsilon_{r}^{''}=2nk$ .

When $n(\tilde{v})$ is close to unity, the band shape for the absorption index is symmetric, like that of the imaginary part of the relative dielectric function. When $n(\tilde{v})$ begins to significantly differ from unity with increasing concentration in the vicinity of the absorption band, $\epsilon_{r}^{''}\left(\tilde{v}\right)$ remains symmetric, while asymmetry begins to appear in the absorption index, as illustrated in Figure 1. The reason for this asymmetry is that the absorption index function is related to the dielectric function and the index of refraction function by $k=\epsilon^{''}/2n$ . Therefore, k is given by Equation (10) (cf. Eq 6):
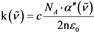



**Figure 1:**
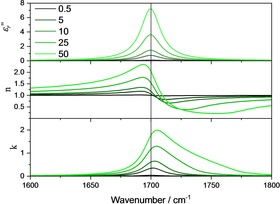
Wavenumber dependence of the imaginary part of the relative dielectric function (upper panel), the index of refraction (center panel) and the index of absorption (lower panel) for different concentrations of 0.5, 5, 10, 25 and 50 mol/l (*S* ^*2^=4900 l/(mol cm^2^), ${\tilde{v}}_{0}=$ 1700 cm^−1^ and *γ*=20 cm^−1^).

and is a function of the index of refraction. According to Beer's law, eqn. (1), absorbance depends on the product *c*⋅*d*. The changes in the shape of the absorbance band with increasing concentration at constant *c*⋅*d* based on eqn. (9) are shown in Figure 2.

**Figure 2:**
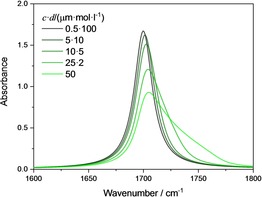
Wavenumber dependence of the absorbance calculated for a model oscillator (*S* ^*2^=4900 l/(mol cm^2^), ${\tilde{v}}_{0}=$ 1700 cm^−1^ and *γ*=20 cm^−1^), with constant value of *c*⋅*d* and concentrations of 0.5, 5, 10, 25 and 50 mol/l.

As the band shape changes, $\overset{*}{\epsilon }\left(\tilde{v}\right)$ clearly changes for constant *c*⋅*d*. Correspondingly, the values for the absorbance at certain wavenumbers vary nonlinearly with concentration. We reported these findings recently.^24^, ^25^ We did not investigate how the area under the curves changes as the band shape changes.

It is well known that if we consider the absorption of a photon as a quantum transition of a harmonic oscillator between two energy levels, then the ratio between the integrated absorbance and the concentration reflects the transition probability.^28^, ^29^, ^30^, ^31^ A linear relation between the integrated absorbance and the concentration indicates that the transition probability does not depend on concentration. Indeed, numerical integration demonstrates such a linear relation, which means that Beer's law holds for the integrated absorbance. Ostensibly, this connection has never been made, probably because Planck's findings were never firmly established in the spectroscopy‐related literature. Even if the connection had been established, it is important to realize the importance of this connection and provide the proper emphasis in the modern literature. The linear dependence of the integrated absorbance is demonstrated in Figure 3. For the neat substance (*c*=50 mol/l), the deviation between the absorbance according to Beer's law and the normalized integrated absorbance is smaller than 0.09 % (range of integration between 100–3000 cm^−1^), where the normalized integrated absorbance is given by Equation 11:
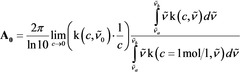



**Figure 3:**
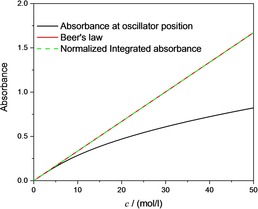
Concentration dependence of the absorbance at the oscillator position (1700 cm^−1^, black curve) compared to the normalized integrated absorbance (green line) and the Lorentz approximation due to eqn. (6) that leads to Beer's law (red line).

where k is calculated from Equation (7) in combination with Equation (9).

Given the form of eqs. (7) and (8), this result is surprising, since it is not clear how integrating the absorption index or the absorbance results in a linear relation between the integrated absorbance and concentration. In fact, it seems that Born faced the same problem when he tried to calculate the “total absorption” of a band for infinitely thin layers by integrating the product of the frequency and the absorption coefficient.^5^ Since he could not find a general solution, he assumed weak absorption. In this case, the integral is as trivial as that of eqn. (4), because the assumption of weak absorption is equivalent to the assumption that eqn. (1) holds strictly. Unfortunately, when Born authored his book,^5^ the Kramers‐Kronig sum rules had not yet been derived.^32^, ^33^, ^34^ One of these sum rules allows us to directly express the result of the integration, apart from a multiplicative constant, as (for the derivation, cf. supporting information):
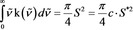



To the best of our knowledge, eqn. (12) has never been used in the context of the (integral) absorbance and its concentration dependence. In the theoretical framework of spectroscopy, integral absorption coefficients are often determined and used, but the fact that linear concentration dependence is only regained by integration of the absorbance is seemingly unknown.^28^, ^29^, ^30^, ^31^ Instead, integral absorbance has been employed to remove the instrumental influence of dispersive spectrometers due to slit functions, and the pointwise validity of Beer's law has enabled the use of integral absorbance.^35^, ^36^, ^37^ Correspondingly, the term “molar oscillator strength” was not generally introduced before refs.^24^, ^25^ defined this quantity.

Note that eqn. (12) has been explicitly derived under the same constraints that apply to Beer's law. In particular, any alteration of the electric field intensity inside the medium must be due to absorption. If the electric field intensity changes locally, e. g., by interference effects (“electric field standing wave effect”),^38^, ^39^, ^40^ scattering, plasmonic enhancement or electromagnetic coupling^[27,41, 42]^ the simple connection between the dielectric function and absorption index is invalidated, and the linear relationship between concentration and (integral) absorbance is therefore revoked. The details of the derivation of the sum rules are discussed in the supporting information and ref.^32^

The employed sum rule (eqn. (12)) can be derived without any reference to a particular oscillator model or band shape function, indicating that it is universal and valid for an arbitrary number of oscillators and bands and in the case of spectral overlap. In the following, we will show that in practice it is not necessary to carry out the integration from zero wavenumber to infinity. For Figure 3, the integration was performed from 100 cm^−1^ to 3000 cm^−1^, but when the integration range was decreased to 1600–1800 cm^−1^, the result was almost unchanged, as shown in Figure 4. At very high concentrations, there is a small deviation from linearity of 0.7 % at *c*=50 mol/l, which is due to the strong asymmetry of the band. This deviation can be drastically decreased to 0.1 % by a small extension of the range of integration to 1840 cm^−1^ (note that this means that for approximately $cS^{*2}/{\tilde{v}}^{2}<0.072$ , eqn. (21) holds and the simplification introduced to derive the sum rule is valid). Thus, the use of the integrated absorbance is error‐tolerant and can therefore be the basis for a robust method. In particular, in spectrophotometry of gases and liquids, baselines are usually not a problem in relation to integration, because the absorbance is employed not as defined, i. e., as the negative decadic logarithm of the incoming and outgoing irradiance, but as the negative decadic logarithm of the transmittance of the solution ratioed to the transmittance of the pure solvent.^43^ This approach removes baselines very effectively. If the cuvettes are thick enough to avoid interference effects,^38^ which is usually the case in the UV/Vis‐spectral range, then the integrated absorbance can be routinely determined.

**Figure 4:**
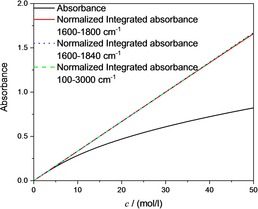
Concentration dependence of the absorbance at the oscillator position (1700 cm^−1^, black curve) compared to the normalized absorbance (green line) integrated from 100–3000 cm^−1^, to the normalized integrated absorbance calculated between 1600–1800 cm^−1^ (red line) and to the normalized integrated absorbance calculated between 1600–1840 cm^−1^ (blue line).

Since the oscillator strength is a conserved quantity and the absorption is therefore very small in certain spectral regions (which do practically not contribute to the integral), we can split the integral in eqn. (12) and integrate over a limited spectral region, provided the chosen limits of the integral ${\tilde{v}}_{a}$ and ${\tilde{v}}_{b}$ are spectral points with negligible absorbance [Eq. 13]:
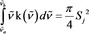



The result is then linearly dependent on the concentration, according to $S_{j}^{2}=c\cdot S_{j}^{*2}$ , where $S_{j}^{*}$ is the molar oscillator strength of a single oscillator or of a number of overlapping oscillators that are well‐separated from the oscillators in the other spectral regions. Eqn. (13) can be seen as a “partial sum rule”.^34^

Note that the sum rules also elucidate another approach to determining the concentration, which is in our opinion an interesting alternative to the approximate calculation of the absorbance via the negative decadic logarithm of the absorbance or reflectance, the (often questionable for solids) correction of the baseline and the determination of the band areas. Instead, the oscillator strength and thus the concentration can, in principle, be determined by dispersion analysis,^9^ which is much more sophisticated than band fitting but is distantly related to it and, to the best of our knowledge, has not been addressed in this context in the literature. Dispersion analysis also takes into account the optical model of the sample (e. g., for liquids in cuvettes: vacuum/cuvette material/liquid layer/cuvette material/vacuum) and automatically corrects the wave‐optics‐related effects, such as interference (“electric field standing wave effects”).^38^, ^39^ In some areas of infrared spectroscopy, dispersion analysis is routinely carried out,^44^, ^45^, ^46^, ^47^ and free and commercial software is available.^48^, ^49^, ^50^ In this sense, the quantity absorbance may eventually become obsolete, and a reformulated Beer's law in integrated form using oscillator strength may supersede it. In addition, dispersion analysis does not require the (corrected) absorbance spectra but can be employed directly for experimental transmittance and reflectance spectra. This approach is possible even for more complex solid samples, such as layered and/or anisotropic materials, for which Beer's law, in general, holds neither in its pointwise nor integral forms, as discussed above.

To summarize, based on a sum rule derived from the Kramers‐Kronig relations, we have reestablished the validity of a modified Beer's law for the special case of isotropic and perfectly homogenous media based on the use of the integrated absorbance. The latter remains linearly dependent on concentration, while the absorbance values at certain spectral points do not necessarily show this linear dependence. Thus, for routine UV‐Vis spectroscopy, the integrated absorbance can be used instead of the peak absorbance to establish calibration curves. For nonroutine use, dispersion analysis can be used as an alternative, which then allows not only the determination of the dispersion parameters but also the concentration of an analyte directly from the transmittance and reflectance spectra.

## Conflict of interest

The authors declare no conflict of interest.

## Supporting information

As a service to our authors and readers, this journal provides supporting information supplied by the authors. Such materials are peer reviewed and may be re‐organized for online delivery, but are not copy‐edited or typeset. Technical support issues arising from supporting information (other than missing files) should be addressed to the authors.

Supplementary
