# Supplementary material for: Beer's Law‐Why Integrated Absorbance Depends Linearly on Concentration
Source: Chemphyschem. 2019 Oct 10;20(21):2748–53. doi: 10.1002/cphc.201900787 (PMC6899465; doi:10.1002/cphc.201900787)
Supplement: Supplementary file 1 — Supplementary [file CPHC-20-2748-s001.pdf]

CHEMPHYSCHEM

Supporting Information

© Copyright Wiley-VCH Verlag GmbH & Co. KGaA, 69451 Weinheim, 2019

Beer's Law-Why Integrated Absorbance Depends Linearly on Concentration

Thomas G. Mayerhöfer,* Andrei V. Pipa, and Jürgen Popp©2019 The Authors. Published by Wiley-VCH Verlag GmbH & Co. KGaA.
This is an open access article under the terms of the Creative Commons Attribution License, which permits use, distribution and reproduction in any medium, provided the original work is properly cited.

The sum rules can be derived based on the Kramers-Kronig relations (KKR).[1-3] One of these sum rules states that the imaginary part of the dielectric function multiplied by the wavenumber is proportional to the squared total oscillator strength:

$$\int_0^\infty \tilde{\nu}\,\varepsilon_r''(\tilde{\nu})\,d\tilde{\nu} = \frac{\pi}{2}S^2\,, \tag{1}$$

To arrive at this rule, we use eqn. (9) of the main document and eliminate in the denominator of the real part the factor $\tilde{\nu}_0^2 - \tilde{\nu}^2$:

$$\varepsilon_r' = 1 + c\frac{S^{*2}}{\tilde{\nu}_0^2 - \tilde{\nu}^2 + \frac{\tilde{\nu}^2\gamma^2}{\tilde{\nu}_0^2 - \tilde{\nu}^2}}\,, \tag{2}$$

Next, we assume a wavenumber $\tilde{\nu}$, (much) higher than the oscillator position, so that $\tilde{\nu}_0^2 << \tilde{\nu}^2$. At this very high wavenumber, we obtain a real value of the relative dielectric function, given by

$$\varepsilon_r' = 1 - c\frac{S^{*2}}{\tilde{\nu}^2}\,, \tag{3}$$

and we can alternatively determine ε_r' from the KKR,[4]

$$1 - c\frac{S^{*2}}{\tilde{\nu}^2} = 1 + \frac{2}{\pi}\wp\int_0^\infty \frac{\varepsilon_r''(\tilde{\nu}')\tilde{\nu}'}{\tilde{\nu}'^2 - \tilde{\nu}^2}d\tilde{\nu}'\,, \tag{4}$$

where $\wp$ indicates the principal value. For the next step, the integral is split into two parts by assuming a wavenumber $\tilde{\nu}_f << \tilde{\nu}$, starting from which the absorption index $\mathrm{k}(\tilde{\nu}_f)$ is virtually zero:

$$1 - c\frac{S^{*2}}{\tilde{\nu}^2} = 1 + \frac{2}{\pi}\wp\int_0^{\tilde{\nu}_f} \frac{\varepsilon_r''(\tilde{\nu}')\tilde{\nu}'}{\tilde{\nu}'^2 - \tilde{\nu}^2}d\tilde{\nu}' + \frac{2}{\pi}\wp\int_{\tilde{\nu}_f}^\infty \frac{\varepsilon_r''(\tilde{\nu}')\tilde{\nu}'}{\tilde{\nu}'^2 - \tilde{\nu}^2}d\tilde{\nu}'\,, \tag{5}$$

Since the second integral is effectively zero and because the higher limit in the first integral is much smaller than $\tilde{\nu}$, we arrive at

$$c\frac{S^{*2}}{\tilde{\nu}^2} = \frac{2}{\pi}\int_0^{\tilde{\nu}_f} \frac{\varepsilon_r''(\tilde{\nu}')\tilde{\nu}'}{\tilde{\nu}^2}d\tilde{\nu}'\,, \tag{6}$$

where we can omit the principal value sign, since the integrand does not diverge. When we multiply both sides by $\tilde{\nu}^2$, we arrive at eqn.(14) of the main manuscript.

A similar sum rule applies to the absorption index, when we consider that

$$\varepsilon_r' = \mathrm{n}^2 = 1 - c\frac{S^{*2}}{\tilde{\nu}^2} \rightarrow \mathrm{n} = \sqrt{1 - c\frac{S^{*2}}{\tilde{\nu}^2}} \approx 1 - c\frac{S^{*2}}{2\tilde{\nu}^2}, \tag{7}$$

At first, it seems paradoxical to assume that eqn. (7) is valid, while eqn. (6) of the main document is generally not. However, if we consider the condition for it to hold, $cS^{*2}/\tilde{\nu}^2 << 1$, and set the oscillator parameters used to generate Figs. 1-3 as constant, then $cS^{*2}/\tilde{\nu}^2 < 0.01$, starting at $\tilde{\nu} > 4950$ cm^{-1}.

Thus, using the result of eqn. (7) and beginning with

$$1 - c\frac{S^{*2}}{2\tilde{\nu}^2} = 1 + \frac{2}{\pi}\wp\int_0^\infty \frac{\mathrm{k}(\tilde{\nu}')\tilde{\nu}'}{\tilde{\nu}'^2 - \tilde{\nu}^2} d\tilde{\nu}', \tag{8}$$

we find, following the same path as for the dielectric function, that

$$\int_0^\infty \tilde{\nu}\,\mathrm{k}(\tilde{\nu})\, d\tilde{\nu} = \frac{\pi}{4}S^2 = \frac{\pi}{4}c \cdot S^{*2}, \tag{9}$$

References

[1] M. Altarelli, D. L. Dexter, H. M. Nussenzveig, D. Y. Smith *Physical Review B*. **1972**, *6*, 4502-4509.
[2] F. Wooten, Optical Properties of Solids, Elsevier Science, **2013**.
[3] D. B. Tanner, Optical Effects in Solids, Cambridge University Press, **2019**.
[4] T. G. Mayerhöfer, J. Popp *Spectrochimica Acta Part A: Molecular and Biomolecular Spectroscopy*. **2019**, *213*, 391-396.
